# Reciprocal regulation of NagC and quorum sensing systems and their roles in *hmsHFRS* expression and biofilm formation in *Yersinia pseudotuberculosis*

**DOI:** 10.1099/mic.0.001397

**Published:** 2023-10-10

**Authors:** Anja Wiechmann, Vanina Garcia, Linzy Elton, Paul Williams, Steve Atkinson

**Affiliations:** ^1^​ Biodiscovery Institute, School of Life Sciences, University of Nottingham, Nottingham, NG7 2RD, UK; ^†^​ Present address: Division of Infection and Immunity, University College, London, WC1E 6BT, UK

**Keywords:** biofilm, *hmsHFRS*, *N*-acetylglucosamine, NagC, quorum, *Yersinia pseudotuberculosis*

## Abstract

Biofilm formation by *Yersinia pseudotuberculosis* is regulated by quorum sensing (QS) and dependent on the haemin storage locus *hms*, required for the extracellular polysaccharide poly-*N*-acetylglucosamine (poly-GlcNAc) production. In *Escherichia coli* NagC regulates both GlcNAc biosynthesis and metabolism with GlcNAc acting as a signal that co-ordinates these and other activities. However, the contribution of NagC and GlcNAc to biofilm development in *Y. pseudotuberculosis* is not known. Here we show that a *Y. pseudotuberculosis nagC* mutant is impaired for biofilm production on abiotic (glass) and biotic (*Caenorhabitis elegans*) surfaces. Genetic complementation restored poly-GlcNAc production and biofilm formation on *C. elegans*. Using *lux*-based promoter fusions, *hmsHFRS* expression was found to be *nagC* dependent. Given that NagC and QS both regulate aggregation and biofilm formation, we investigated their regulatory relationship using *lux*-based promoter fusions. These revealed that (i) *nagC* is negatively autoregulated, but expression can be partially restored in the *nagC* mutant by exogenous GlcNAc, (ii) NagC negatively regulates the *ytbI* and *ypsI* QS genes and (iii) *nagC* expression is reduced in the *ytbI*, *ypsI* and *ypsR* mutants but not the *ytbR* mutant. These data establish the existence of a reciprocal regulatory relationship between NagC and QS, which in the case of the *luxRI* pair *ytbRI*, is also GlcNAc-dependent. NagC and GlcNAc are therefore components of a regulatory system involving QS that modulates biofilm formation and aggregation.

## Introduction

*Yersinia pseudotuberculosis* is a highly adaptable psychrotrophic pathogen which, in humans causes self-limiting enterocolitis but can adapt to survive in a range of hosts and external environments. Part of this successful survival strategy relies on the production of a self-generated extracellular polymeric (EPS) adhesive matrix, usually referred to as a biofilm [1]. It has been known for some time that *Y. pseudotuberculosis* and its near identical [2] relative *Y. pestis* form biofilms on biotic surfaces such as the cuticle of the nematode *Caenorhabditis elegans* [3, 4] and in *Y. pestis,* on the proventriculus and in the gut of the oriental rat flea *Xenopsylla cheopis* [5, 6]. Some *Y. pseudotuberculosis* strains can also form biofilms on abiotic surfaces [4]. Using specific antibodies or the lectin wheat germ agglutinin (WGA), the major extracellular polysaccharide present in *Yersinia* EPS has been shown to be poly-GlcNAc, the polymeric form of the amino sugar β−1,6-*N*-acetyl-d-glucosamine (GlcNAc) [7–9]. This is synthesised and exported by components of the haemin storage loci (*hmsHFRS*, *hmsCDE*, *hmsT*, *hmsP*, *hmsB*) [3, 10–16]. The role of the *hms* locus is well documented with mutations in the *hmsHFRS* genes resulting in reduced *Y. pseudotuberculosis* and *Y. pestis* biofilm formation on the surface of *C. elegans* [3] while flea proventricular and gut blockage are also attenuated in *Y. pestis hms* mutants [8, 9, 16–19].

In *Y. pseudotuberculosis,* quorum sensing (QS) depends on two pairs of LuxRI orthologues termed YpsRI and YtbRI which are responsible for the production (YpsI and YtbI) and sensing (YpsR and YtbR) of a range of *N*-acyl homoserine lactones (AHLs) [20]. These include *N*-hexanoyl homoserine lactone (C6-HSL), *N*-(3-oxohexanoyl) homoserine lactone (3-oxo-C6-HSL), *N*-octanoyl homoserine lactone (C8-HSL), *N*-(3-oxooctanoyl) homoserine lactone (3-oxo-C8-HSL) and *N*-(3-oxodecanoyl) homoserine lactone (C10-HSL). AHL-dependent QS in *Y. pseudotuberculosis* is organized hierarchically such that the YpsRI system negatively autoregulates itself while positively regulating the expression of the *ytbRI* system. Conversely*,* the YtbRI system is positively autoregulated but only at the level of *ytbI* expression and does not regulate *ypsR* or *ypsI* [20]. AHL production in *Y. pseudotuberculosis* is abolished when both *ypsI* and *ytbI* are mutated but not after deletion of both *ypsR* and *ytbR* indicating that, as in other *Yersinia* species*,* AHL production is not dependent on the presence of a functional YpsR or YtbR protein [21, 22].

In addition to controlling, flagella-mediated motility, cell aggregation and type three secretion (T3S), QS in *Y. pseudotuberculosis* also regulates biofilm formation on *C. elegans* in a T3S system-dependent manner [22, 23]. *Y. pseudotuberculosis* biofilms on *C. elegans* contain AHLs and mutation of both *ypsI* and *ytbI* or both *ypsR* and *ytbR* severely reduced biofilm formation [23]. However, curing the pYV virulence plasmid which carries the T3S system genes or mutating the type T3S structural component gene *yscJ* in a *ypsI ytbI* double mutant restored biofilm formation [23].

Aside from being a component of *Yersinia* spp. biofilms, GlcNAc has multiple roles in nature and is required for bacterial cell wall peptidoglycan and in its polymeric form as chitin, it is also essential for fungal cell walls, crustacean and arthropod exoskeletons. It is therefore unsurprising that most bacterial species possess orthologues of the *Escherichia coli nagE-nagBACD* which are responsible for the uptake of GlcNAc and its degradation to fructose-6-phosphate. In *E. coli* GlcNAc (as intracellular GlcNAc-6P) availability determines its own synthesis or breakdown through the activity of the ROK (Repressors, Open reading frames and Kinases) family protein, NagC [24–26], which represses the expression of *nagE-nagBACD* in the absence of intracellular GlcNAc-6-P, the product of periplasmic NagE kinase activity on GlcNAc [20, 27]. Increasing levels of extracellular GlcNAc will therefore result in de-repression of the *nag* operon. NagC also acts as an activator and repressor of the *glmUS* operon, the products of which synthesise GlcNAc in a GlcNAc-6P dependent manner [25].

*Y. pseudotuberculosis* NagC is approximately 78 % orthologous to the *E. coli* NagC and is therefore assumed to perform a similar function although to our knowledge no role has yet been assigned. Because *Y. pseudotuberculosis* and *Y. pestis* biofilms are known to contain poly-GlcNAc, and given the regulatory links between QS and biofilms, we investigated the role of NagC in *Y. pseudotuberculosis *. Here we show that a *nagC* mutant is impaired for biofilm formation in *C. elegans* infection models and on glass while exogenous GlcNAc restored biofilm formation. We also found that *nagC* is autoregulated, positively controls the *hms* locus and negatively impacts on QS. We also establish the existence of reciprocal regulatory relationship between NagC and QS.

## Methods

### Bacterial strains, plasmids and growth conditions

The *Y. pseudotuberculosis* YPIII pIB1, *E. coli* and *C. elegans* strains have been described elsewhere unless otherwise stated [23]. Plasmids and primers used in this study are listed in Tables S1 and S2 respectively. *Y. pseudotuberculosis* was routinely grown in YLB and unless otherwise stated growth conditions, media, DNA manipulations and analysis were as previously described [22, 23]. *C. elegans* and biofilm assays were performed at 22 °C to ensure *C. elegans* were at their optimum growth conditions but due to the limitations of the Tecan GeniusPro luminometer/spectrophotometer (described below) all promoter fusion assays were performed at 26 °C (rather than 22 °C) and 37 °C.

### Construction of a *Y. pseudotuberculosis nagC* mutant and the complemented mutant

An in-frame deletion of *nagC* was constructed by inserting a kanamycin cassette [23] between base 60 and base 1176 of the 1267 bp *nagC* ORF using the λ-red recombinase system [28, 29]. A 1029 bp kanamycin cassette from pUC4K was amplified (*nagC*-ko-F and *nagC*-ko-R) incorporating flanking regions of *nagC* −9 to −60 bp before the start and after the stop codons respectively. The PCR product was transformed into *Y. pseudotuberculosis* YpIII containing the λ-red recombinase helper plasmid pAJD434. After growth on arabinose to induce recombination and antibiotic selection, temperature sensitive pAJD434 was removed at 37 °C and the recombinants confirmed by PCR.

To genetically complement the *nagC* mutant a 1817 bp PCR product (primers *nagC*-comp-F and *nagC*-comp-R) was cloned into pGEM-T Easy and the fragment was excised (XhoI/BamHI) and cloned into pUC18R6KT::Tc mini Tn*7* (to give pAW1) to enable the targeted insertion of functional *nagC* into the chromosome at the Tn*7 glmS*/*pstS* site [30] rather than rely on a plasmid borne copy of *nagC*. The transposase helper vector pTNS2 and pAW1 were co-transformed into the *Y. pseudotuberculosis nagC* mutant and transformants were screened by PCR to confirm that the functional copy of *nagC* had integrated downstream of *glmS*.

### Construction and analysis of *lux*-based promoter fusions

For construction of a *nagC* promoter *luxCDABE* fusion, the 508 bp promoter region of *nagC* was amplified (*nagC-lux*-F and *nagC-lux*-R) and the PCR product was cloned into pGEMT/Easy. A KpnI/BamHI *luxCDABE* cassette was excised from pBlue*lux* [22] and cloned immediately downstream, and the resulting P*_nagC::lux_* promoter fusion was excised as an XhoI/SphI fragment and cloned into pDM4 to give pDM4::P*_nagC_*_::*lux*_. P*_nagC_*_::*lux*_ was then conjugated into *ypsI*, *ypsR*, *ytbI*, *ytbR* [22] and the newly constructed *nagC* mutants. Our QS gene promoter fusions [pHP276 (P*_ypsR_::lux*), pHP277 (P*_ypsI_::lux*), pHP278 (P*_ytbR_::lux*) and pHP279 (P*_ytbR_::lux*)] [22] were also conjugated into the *Y. pseudotuberculosis* parent, *nagC* mutant and the complemented *nagC* mutant.

To construct the *hmsHFRS* promoter fusion to *luxCDABE* a 687 bp PCR product upstream to *hmsH* (primers VG-Phms-F2-ApaI and VG-Phms-R-XhoI) was cloned into pBlue*lux* [22] as an ApaI/XhoI fragment. The resulting P*_hms_*_::*lux*_ was cloned into pDM4 as an ApaI/SpeI fragment for conjugation. Unfortunately, the bioluminescent signal was too low for detection and therefore the vector was linearized (ApaI), Klenow treated and the cassette excised with BamHI and cloned (SmaI and BamHI) into the low copy number vector pHG327 to give pHG::P*_hms_*_::*lux*_ which was cloned into the *nagC* mutant and its complement.

Lux-based reporter fusion assays were performed by diluting overnight cultures (30 °C) 1 : 100 and re-growing to mid-log phase, washed twice and diluted to OD_600_ of 0.006. Then 300 µl samples were transferred (×3) into a 96-well microtitre plate and luminescence as relative light units (RLU) and OD measurements were taken every 30 min for 18 h at 37 °C or 26 °C using a Tecan GeniusPro luminometer/spectrophotometer. The expression profiles were plotted as RLU/OD against time and the curves were used to calculate the area under the curve (AUC) for each strain [31]. For simplicity AUC is presented in the results and the full expression profiles which were used to generate the AUC are shown in the supplementary data.

### AHL extraction and analysis

AHL analysis was performed using an adapted protocol described by [32]. The *Y. pseudotuberculosis* parent, *nagC* mutant and the genetically complemented mutant were grown in 5 ml YLB-MOPs (50 mM MOPS, pH 6.8) to an OD_600_ of 1.0 at 37 °C. Cells were harvested and 1 ml of cell free supernatant was mixed with an internal standard (d9-C5-AHL) at a final concentration of 0.1 µM. The deuterium component of the internal standard allows differentiation from C5-AHL by mass spec and allows the resulting concentration to be used as a control marker. Then 0.5 ml of 0.1 % acetic acid in ethyl acetate was added to the supernatant before the sample was vortexed for 30 s and centrifuged at 2000 ***g*** for 5 min. The upper organic layer containing solubilised AHLs was pooled in a fresh tube and the process repeated twice more on the lower inorganic layer. The pooled sample was then dried using a centrifugal evaporator and resuspended in 10 µl acetonitrile.

AHL analysis was conducted using a modified version of the method reported by Ortori *et al*., (2011) [32] whereby liquid chromatography (LC) was performed using an Excion LC, featuring a binary gradient AD pump system and a Phenomenex Gemini C18 column (3.0 µm, 100×3.0 mm) with an appropriate guard column. The column oven and autosampler were maintained at 40 and 5°C respectively. Mobile phase A was 0.1 % (v/v) formic acid in water, and mobile phase B 0.1 % (v/v) formic acid in methanol. The flow rate throughout the chromatographic separation was 450 µl min^−1^. After a 5 µl sample injection, the binary gradient increased from 0 % B to 40 % B over 0.5 min. This then increased further to 99 % B over 6.5 min. After 1 min at this composition, percent B decreased to 0 over 0.1 min and remained at this composition for another 1.9 min with a total run time per sample of 10 min. The mass spectrometry (MS) system was an AB Sciex Instruments Qtrap 6500+ hybrid triple-quadrupole linear ion trap mass spectrometer, equipped with an electrospray ionisation (ESI) interface. Instrument control, data collection and analysis were conducted using Analyst software (v 1.7.3). Source parameters were set as: curtain gas: 20.0, ion source potential: 5500 V, source temperature: 450 °C, GS1 : 50.0, and GS2 : 50.0. AHL Synthetic standards were synthesised in house and dried, extracted samples were stored at −20 °C. Prior to analysis, each sample extract was redissolved in 50 µl of MeOH. LC-ESI-MS/MS methods were used for the analysis of AHLs in bacterial supernatant extract samples. Analysis was conducted with the MS operating in MRM (multiple reaction monitoring) mode, screening the LC eluent for the specific analytes of interest.

### *C. elegans* and glass biofilm assays

Routine culturing of *C. elegans*, biofilm infection assays, calculation of the biofilm severity index (BSI) and confocal laser scanning microscopy (CLSM) were performed as previously described [4, 23, 33]. The lectin Wheat Germ Agglutinin (WGA) binds to the GlcNAc component of *Yersinia* spp. biofilms [7] and when conjugated to Rhodamine (Rho-WGA) can be used as a red fluorescent label to stain biofilms on the surface of *C. elegans* or on cells grown in planktonic cultures [23]. *Y. pseudotuberculosis* were fluorescently labelled by transforming with pSB2019 [34] which constitutively expresses Gfp.

To culture biofilms on glass each strain was grown in *Yersinia* defined minimal media (YDMM) (1 x M9 minimal salts (Gibco), 0.4 % glucose, 0.4 % casamino acids, 10 mM MgCl2, 5 mM K_2_SO_4_ and 38 µM Thiamine) [35] and after washing to an OD_600_ of 0.5 in freshly prepared YDMM 1 ml was transferred (×3) to a 24-well glass bottomed micro-titre plate (Greiner, BioOne), sealed with plastic film and statically incubated for 24 h. The wells were washed twice (dH_2_O), air dried and the biofilm was stained with 300 µl of 5 μg ml^−1^ wheat germ agglutinin (WGA) conjugated to fluorescein (Flu-WGA) (Vector Laboratories). Plates were then covered and incubated at 4 °C for 2 h and washed twice with sterile dH_2_O and air-dried before quantitative analysis of total biofilm production was performed by recording the absolute fluorescence (484 nm absorption, 512 nm emission) of a 15×15 circular scan area (omitting the outer 2.0 mm) with a Tecan SPARK plate reader.

### PolyGlcNAc detection in planktonic *Y. pseudotuberculosis*

For Rho-WGA labelling a modified method described by Yoong *et al*. [36] was performed in which 40 µl of an overnight planktonic culture was spotted onto a microscope slide and allowed to air dry. Pre-diluted (1/1000) Rho-WGA (20 µl) was added to the dried cells and incubated at room temperature in the dark for 45 min before being examined by CLSM. Each CLSM image was then analysed using the Volocity 2.0 (Perkin Elmer) software package in which each image was measured and analysed using the mean of the sum fluorescence.

## Results

### The *Y. pseudotuberculosis nagC* mutant produces less poly-GlcNAc at 37°C compared with 22°C

**Fig. 1.**
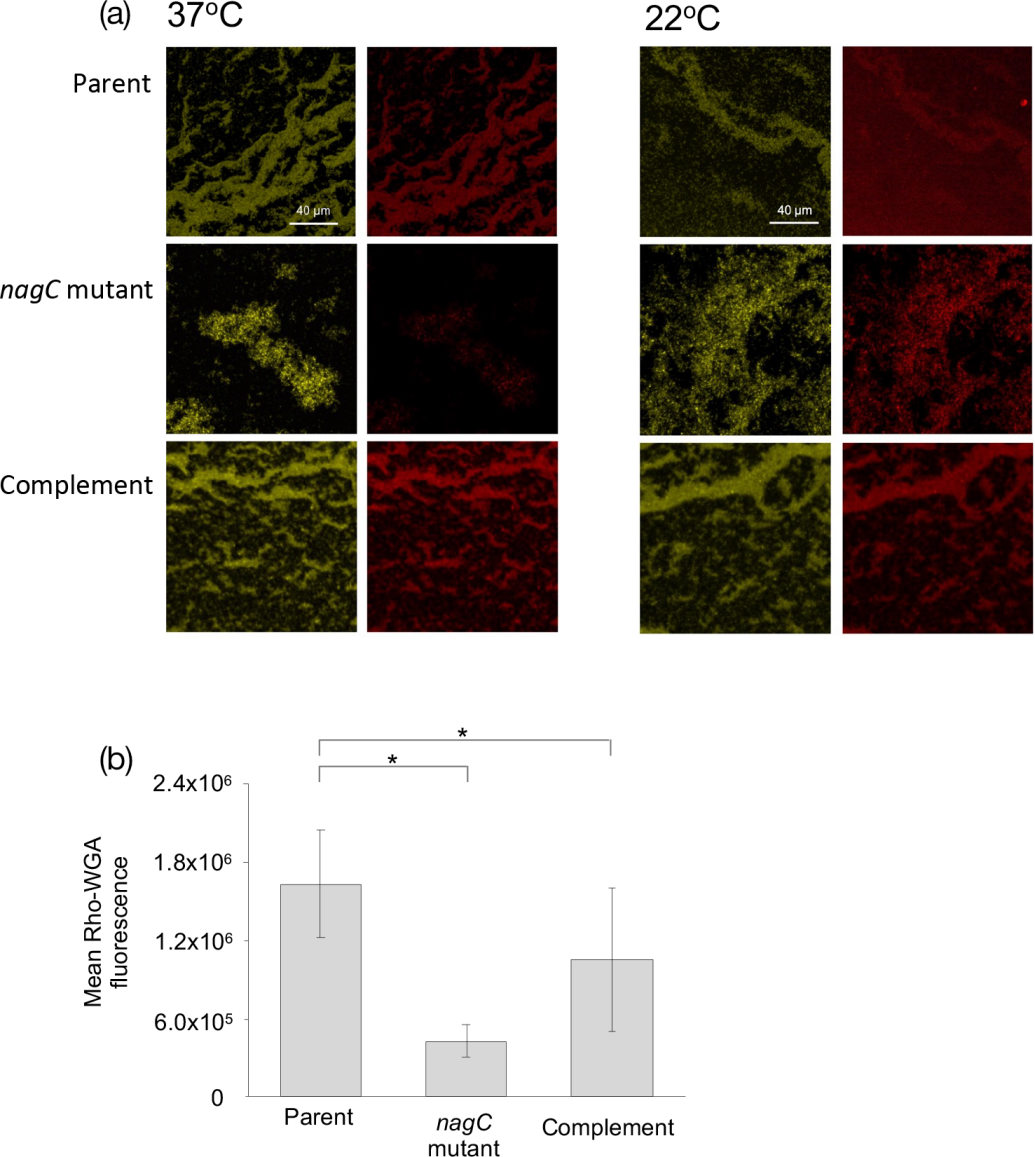
NagC regulates Poly-GlcNAc production on planktonic *Y. pseudotuberculosis* cells at 37 °C. CLSM revealed that there was less Rho-WGA staining of poly-GlcNAc in planktonic cultures in the Gfp-labelled *nagC* mutant when compared to the parent at 37 °C. Left panels, Gfp-labelled *Y. pseudotuberculosis *; right panels, Rho-WGA staining of poly-GlcNAc (**a**). When the CLSM images were quantified, a similar pattern emerges with mean fluorescence as a measure of biofilm reduced in the *nagC* mutant when compared to the parent and complement (**b**) (*n*=3 *P=<0.01).

Following construction and complementation of the *nagC* mutant, poly-GlcNAc production in planktonic cells was examined at 37 and 22 °C in a Gfp-labelled *Y. pseudotuberculosis* parent, *nagC* mutant and genetically complemented *nagC* mutant grown overnight in the presence of Rho-WGA. Fig. 1a shows the presence of Gfp-labelled *Y. pseudotuberculosis* and that the amount of red fluorescence as a marker of poly-GlcNAc was severely reduced in the *nagC* mutant at 37 °C when compared with the parent and complemented mutant. There were no differences at 22 °C. When the 37 °C confocal images were quantified, the mean fluorescence was reduced greater than three-fold in the *nagC* mutant when compared with the parent (Fig. 1b).

### NagC regulates biofilm formation on biotic and abiotic surfaces

**Fig. 2.**
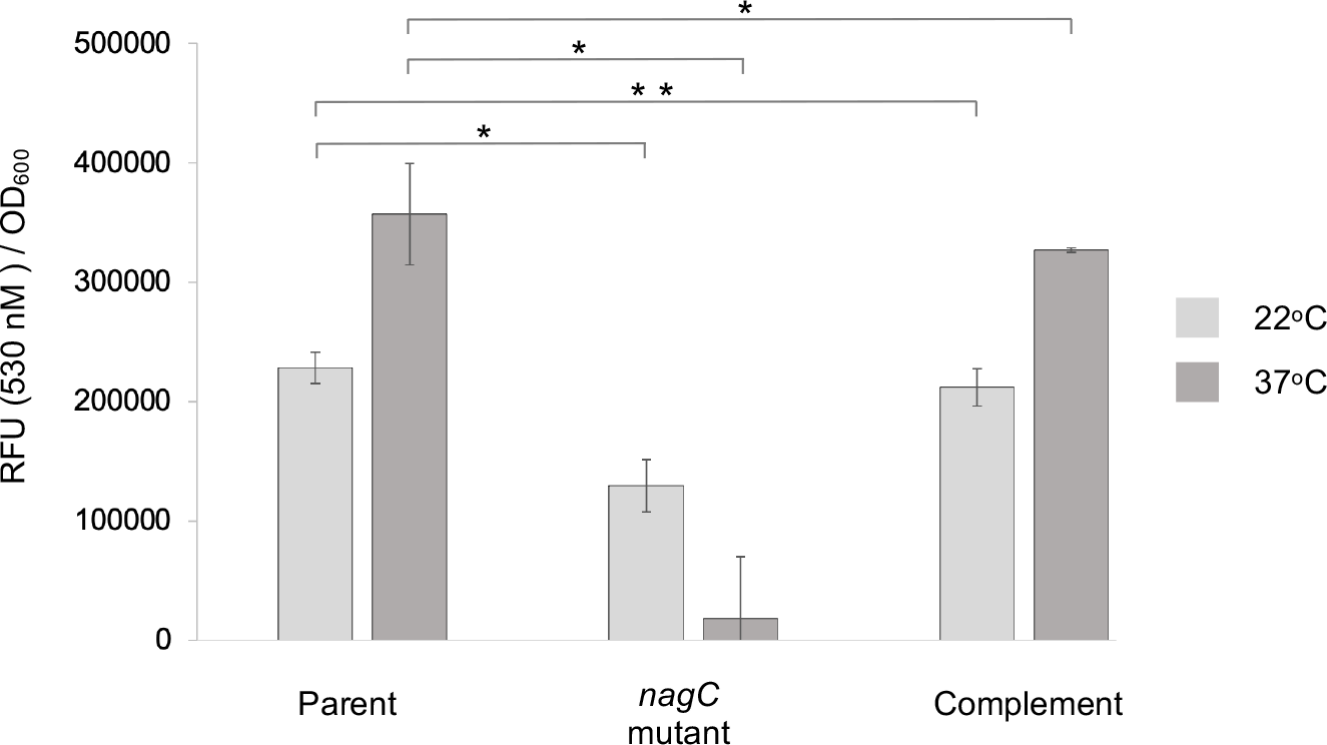
Biofilm formation on glass is NagC dependent. When the *Y. pseudotuberculosis* parent, *nagC* mutant and complement were grown at 37 °C on glass and Rho-WGA fluorescence quantified as a measure of biofilm biomass, the *nagC* mutant biofilm was reduced but was restored to levels similar to the parent in the genetically complemented strain. Although the trend remained the same, the difference was less evident between the parent and mutant at 22 °C (*n*=3 *P=<0.005 **P=<0.01).

Since NagC positively regulated poly-GlcNAc biosynthesis at 37 °C we surmised that mutation of *nagC* may result in reduced biofilm formation. In glass surface biofilm assays, there was a ~15 fold reduction in biofilm (as quantified by Flu-WGA fluorescence) at 37 °C in the *nagC* mutant when compared with the parent and complemented mutant whereas at 22 °C the reduction was less only ~2-fold with the overall levels much reduced when compared to 37 °C (Fig. 2).

**Fig. 3.**
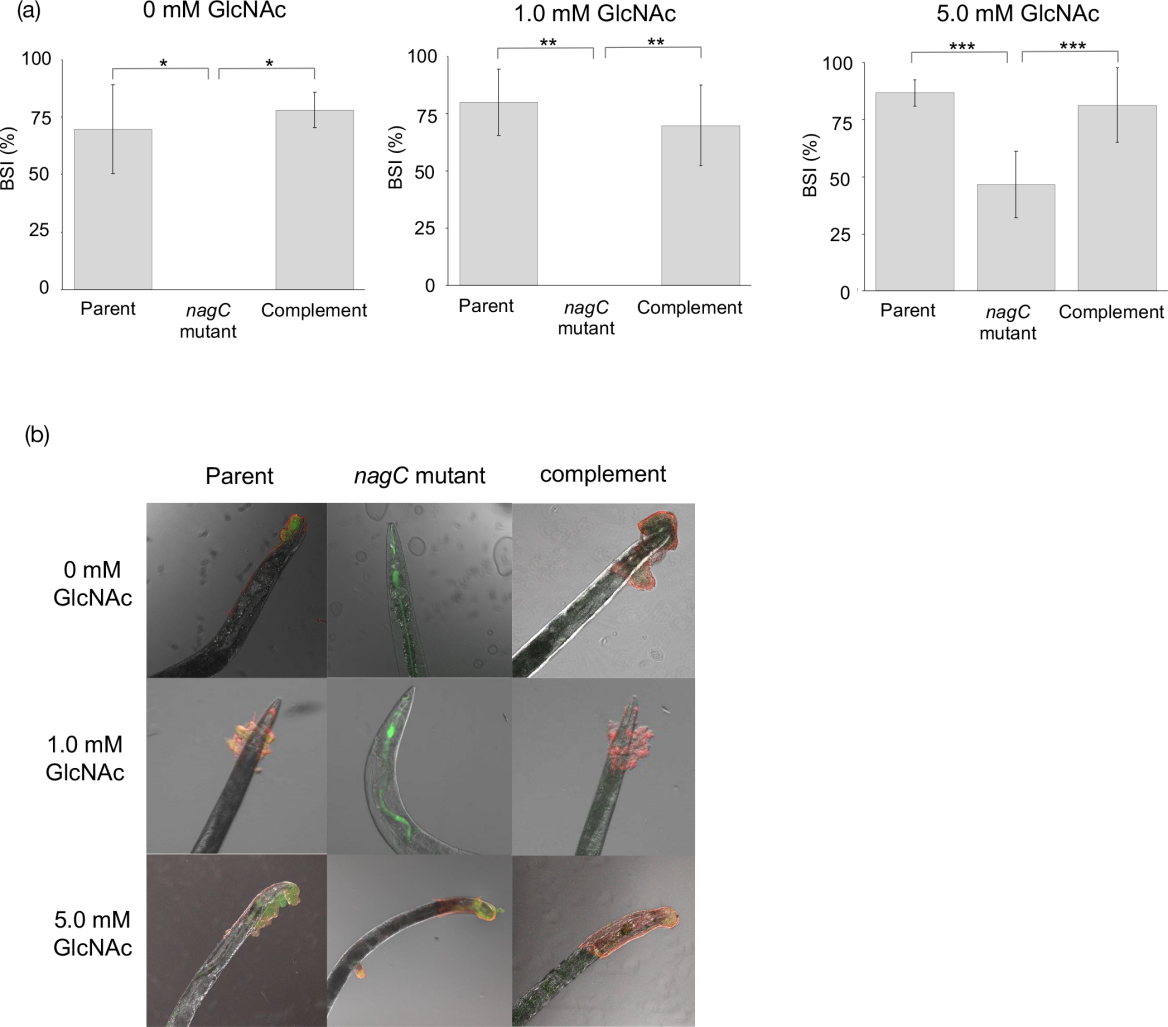
Phenotypic assays revealed NagC regulates biotic biofilm formation on *C. elegans* and aggregation in planktonic cultures. The biofilm severity index (BSI) revealed that biofilm formation was severely attenuated in the *nagC* mutant when compared to the parent in the absence or presence of 1.0 mM GlcNAc. However, in the presence of 5.0 mM GlcNAc biofilm formation was partially restored in the *nagC* mutant and in all cases biofilm was restored to levels comparable to the parent in the complemented *nagC* mutant strain. (**a**). CLSM revealed that when the biofilm was stained red with rhodamine labelled WGA, green *Y. pseudotuberculosis* expressing Gfp were seen embedded in the red biofilm matrix (composite orange) in the parent, considerably reduced in the *nagC* mutant but restored in the complemented strain. Adding 1.0 mM GlcNAc had no effect but the addition of 5 mM GlcNAc restored biofilm formation in the *nagC* mutant (**b**) (*n*=3 *P=<0.005, **P=<0.001, ***P=<0.01).

In *C. elegans* infection assays, when compared with the parent and complemented *nagC* mutant, the biofilm severity index (BSI) as a measure of biofilm formation, was almost abolished in the *nagC* mutant (Fig. 3a). This was also the case in the presence of 1.0 mM GlcNAc but when 5.0 mM GlcNAc was added to the *nagC* mutant, biofilm was restored to approximately 50 % of that produced by the parent and complemented mutant (Fig. 3a). *C. elegans* infected with GFP-labelled *Y. pseudotuberculosis* grown in the presence/absence of GlcNAc were stained with Rho-WGA and examined by confocal laser scanning microscopy (CLSM). Fig. 3b shows the green fluorescent parent and the *nagC* complemented strain were embedded in a red fluorescent Rho-WGA labelled biofilm matrix which was severely reduced on worms that had been infected with the *nagC* mutant (Fig. 3b; 0 mM GlcNAc). A similar pattern emerged when *C. elegans* was infected in the presence of 1.0 mM GlcNAc but when increased to 5.0 mM, the *nagC* mutant formed a biofilm on the worm surface that was similar to that produced by the parent *Yersinia* strain (Fig. 3b).

### NagC regulates *hmsHFRS* expression

**Fig. 4.**
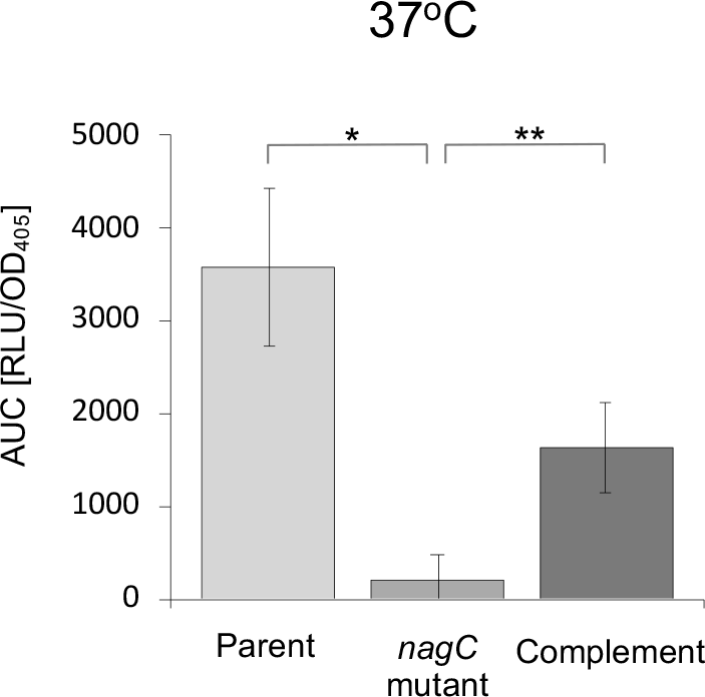
Expression of *hmsHFRS* in the *nagC* mutant. Using a *lux*-based promoter fusion, at 37 °C *hmsHFRS* expression was reduced approximately 12-fold when compared to the parent and genetic complementation of the *nagC* mutant partially restored expression the levels seen in the parent (*P=<0.001, **P=<0.01).

Since *hmsHFRS* is required for poly-GlcNAc production and hence biofilm formation, we wanted to determine whether NagC was involved in the regulation of *hmsHFRS*. A low copy plasmid containing an *hmsHFRS lux*-based promoter fusion (pHG::P*_hms_*_::*lux*_) was constructed and transformed into the parent, *nagC* mutant and complemented mutant. Fig. 4 shows that at 37 °C the expression of *hmsHFRS* was reduced by ~12-fold in the *nagC* mutant when compared with the parent with the complemented mutant partially restoring levels back to those of the parent suggesting that NagC acts as an activator of *hmsHFRS* at this temperature. At 26 °C a similar trend emerges with a small but not significant reduction (<2-fold) in the expression of the *nagC* mutant compared with the parent (data not shown).

### *nagC* is negatively autoregulated by NagC and expression is controlled by GlcNAc in a dose-dependent manner

**Fig. 5.**
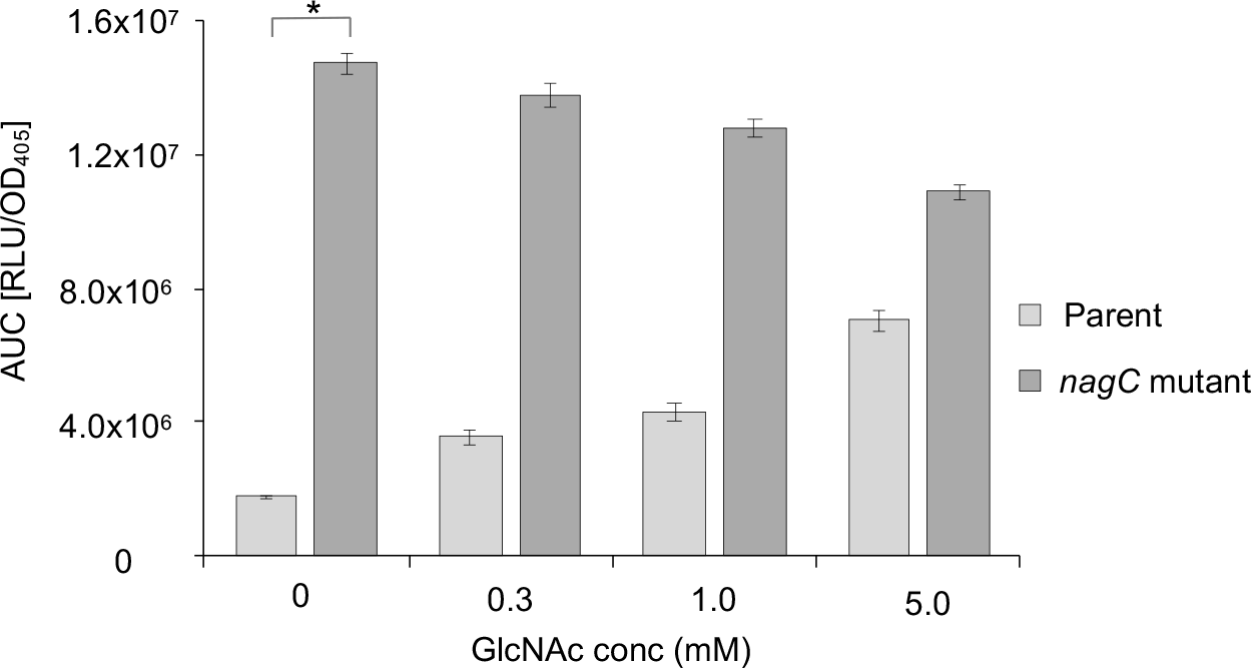
*nagC* is negatively autoregulated by NagC and expression controlled by GlcNAc in a dose-dependent manner. *nagC* expression increased with increasing concentrations of GlcNAc (0, 0.3, 1.0, 5.0 mM) at 37 °C in the parent, whereas expression remains similar when compared to the control (0 mM) in the *nagC* mutant (*n=*3 *P=<0.0001).

We found that the upstream region of *Y. pseudotuberculosis nagC* possesses −10 and −35 sequences as well as a putative *nagC* binding site with only two mismatches to the consensus [37] (Fig. S1A, available in the online version of this article), which prompted us to construct a *nagC* promoter fusion (P*_nagC_::lux*) to this region which was integrated into the parent and *nagC* mutant. At 37 °C *nagC* expression increased approximately eight-fold in the absence of GlcNAc in a *nagC* mutant background when compared with the parent suggesting NagC can repress its own expression (Fig. 5, 0 mM GlcNAc). *nagC* expression in the parent then increased in a dose dependent manner following the addition of 0.3 mM, 1.0 mM and 5.0 mM GlcNAc suggesting that *nagC* expression is also subject to the same GlcNAc-dependent de-repression that is seen in *E. coli *. However, the addition of GlcNAc to the *nagC* mutant had little effect on *nagC* expression which remains comparable with the GlcNAc-free control (Fig. 5). At 26 °C a similar pattern emerged although the overall levels of expression were approximately five-fold lower (data not shown). The expression profiles over 18 h from which the AUC data was generated are shown in Fig. S1 B-E.

### NagC regulates the *Y. pseudotuberculosis* quorum sensing system in a temperature dependent manner

**Fig. 6.**
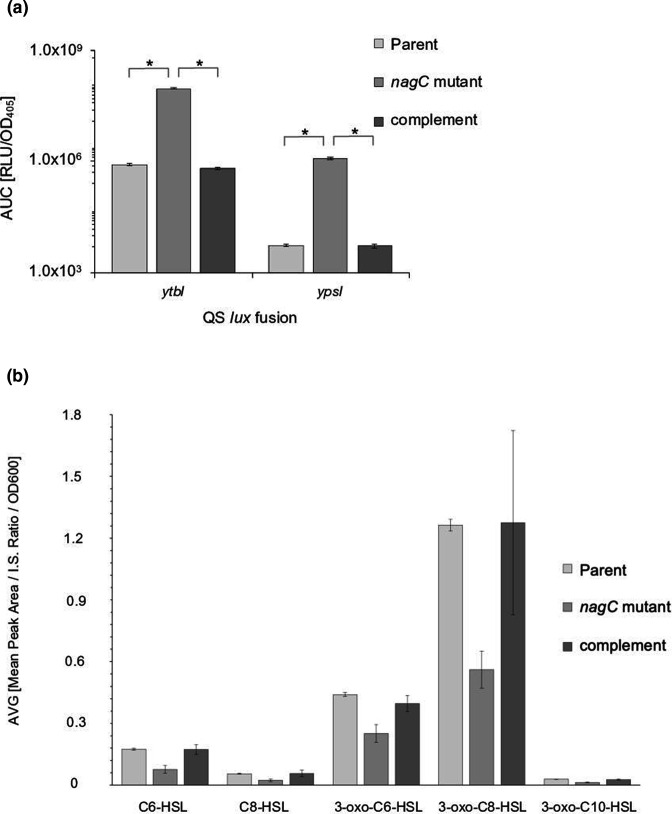
NagC regulates the *Y. pseudotuberculosis* AHL synthase gene expression but not AHL production. At 37 °C expression of *ytbI* and *ypsI* were upregulated in the *nagC* mutant when compared to the parent but downregulated to parental levels when genetically complemented with *nagC*. (**a**) (*n*=3 *P=0.0001). In contrast to the highly up-regulated expression of the two AHL synthase genes, when AHLs were extracted from cell-free supernatants from the *Y. pseudotuberculosis* parent, the *nagC* mutant and its complement, LC-ESI-MS/MS revealed that there was an ~50 % reduction in the concentration of the major AHLs in the *nagC* mutant when compared to the parent. AHL levels were restored to those obtained in the parent when the *nagC* mutant was complemented with a functional copy of *nagC* (**b**).

Given the role of NagC as a repressor of GlcNAc metabolism, the contribution of poly-GlcNAc to the composition of biofilms and our previous observations showing that QS and biofilm formation on *C. elegans* are interdependent [23] suggested that there may be a regulatory relationship between NagC and the *Y. pseudotuberculosis* QS system. To investigate this at the transcriptional level, *lux*-based fusions (P*_ypsI_::lux*, P*_ypsR_::lux*, P*_ytbI_::lux* and P*_ytbR_::lux* [22]) were introduced as single copies into the *Y. pseudotuberculosis* parent, *nagC* mutant and complemented *nagC* mutant. At 37 °C *ytbI* and *ypsI* expression increased up to ~120-fold and ~300-fold respectively in the *nagC* mutant when compared to the parent and complemented *nagC* mutant (Fig. 6a) and a similar trend was observed at 26 °C although the overall expression levels were lower (data not shown). These data suggest that at both temperatures *ytbI* and *ypsI* expression are negatively regulated by NagC. There was no appreciable change in *ypsR* or *ytbR* expression in the *nagC* mutant when compared with the parent at either temperature (data not shown). The expression profiles over 18 h from which the AUC data was generated are shown in Fig. S2A–D.

We surmised that given the elevated levels of AHL synthase expression, this may result in an increase in the quantity of signal molecule produced by the *nagC* mutant and we therefore extracted AHLs from culture supernatants taken from the *Y. pseudotuberculosis* parent, *nagC* mutant and the complemented *nagC* mutant. LC-ESI-MS/MS revealed that, contrary to expectations, the concentrations of the major *Y. pseudotuberculosis* AHLs, namely C6-HSL, C8-HSL, 3-oxo-C6-HSL, 3-oxo-C8-HSL and 3-oxo-C10-HSL were each reduced by ~50 % in cell-free supernatant extracts taken from the *nagC* mutant when compared to the parent and the *nagC* mutant complement grown under the same conditions used for the Lux-based assays (Fig. 6b).

### GlcNAc influences the expression of *ytbI* in a NagC-dependent manner

**Fig. 7.**
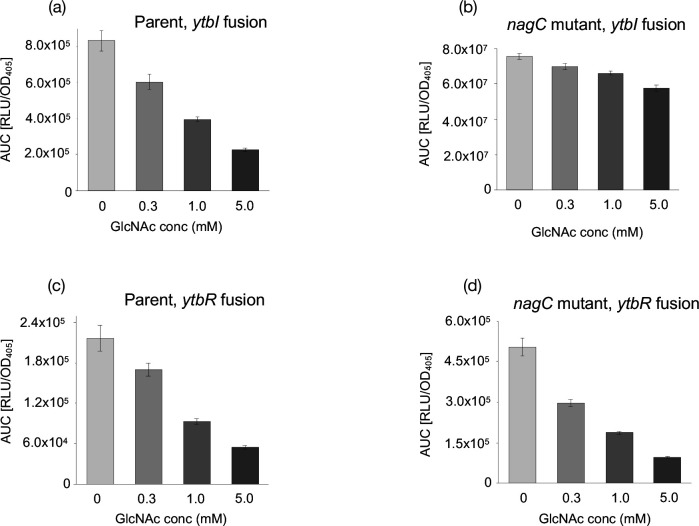
Expression of QS genes in the *nagC* mutant in 0, 0.3, 1.0 and 5.0 mM GlcNac at 37 °C. The expression of *ytbI* and *ytbR* was reduced in a dose dependent manner in the parent (**a and c**) while *ytbR* expression decreased in a similar manner in the *nagC* mutant (**d**) but was relatively unresponsive to GlcNAc concentration in the *ytbI* fusion (**b**).

Since *ytbI* and *ypsI* expression appear to be repressed by NagC (Fig. 6a) we examined whether GlcNAc could also influence QS gene expression. The parent, *nagC* mutant and complemented mutant carrying the *lux*-based *ytbI* and *ytbR* promoter fusions were grown at 37 °C in 0 mM, 0.3 mM, 1.0 mM and 5.0 mM GlcNAc, which revealed that exogenous GlcNAc reduced the expression of *ytbI* in a dose dependent manner in the parent (Fig. 7a) whereas the *nagC* mutant was considerably less responsive (Fig. 7b). Increasing concentrations of GlcNAc also reduced the expression of *ytbR* but in this case the same trend was observed in both the parent and *nagC* mutant (Fig. 7c, d). These data suggest that *ytbI* and *ytbR* expression are GlcNAc dependent but *ytbI* expression requires the presence of NagC. The expression profiles over 18 h are shown in Fig. S3A–D. In contrast, there was little change in expression of *ypsI* or *ypsR* at 37 °C in the parent or *nagC* mutant in the presence of GlcNAc (Fig. S4A–D). The expression profiles over 18 h are shown in Fig. S4E–H. A similar dose response to that observed at 37 °C was also noted at 26 °C for *ytbI* and *ytbR* in the parent and *nagC* mutant (Fig. S5A–D). However, *ypsI* or *ypsR* expression in the parent or *nagC* mutant showed no response to exogenous GlcNAc (Fig. S5E–H). The expression profiles over 18 h are shown in Fig. S6A–H.

### The *Y. pseudotuberculosis* QS system reciprocally regulates *nagC* expression

**Fig. 8.**
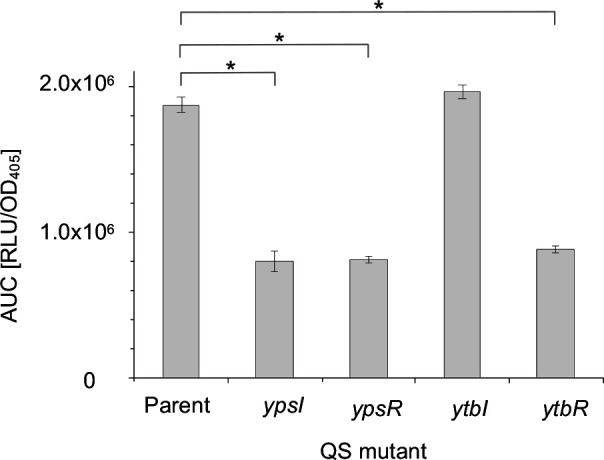
Expression of *nagC* in the QS mutants at 37 °C. *nagC* expression was reduced in the *ypsI, ypsR* and *ytbR* mutants whereas expression remained similar to the parent in the *ytbI* mutant (*n*=3 *P=<0.001).

After confirming that GlcNAc influences *nagC* expression and NagC regulates QS gene expression we investigated whether there was a reciprocal regulatory relationship between the QS system and NagC. *nagC* expression was examined in the parent and QS mutants containing P*_nagC_::lux* at 37 and 26 °C. At 37 °C *nagC* expression was reduced in the *ypsI, ypsR* and *ytbR* mutants when compared to the parent suggesting QS activates *nagC* expression (Fig. 8), with a very similar pattern emerging at 26 °C although the overall expression levels were higher (data not shown). The expression profiles at 37 °C are shown in (Fig. S7).

## Discussion

Poly-GlcNAc is a key component of *Y. pseudotuberculosis* and *Y. pestis* biofilms at 37 °C and given that reduced Rho-WGA labelling of the *nagC* mutant was observed these data suggested that there was a link between NagC and poly-GlcNAc production. It is however noteworthy that there is little or no equivalent reduction in poly-GlcNAc at 22^ ^°C. Temperature and strain variation linked to virulence may affect poly-GlcNAc formation as reported by Yoong *et al*. [36] who showed that when compared with non-pathogenic virulence plasmid negative strains, biofilms formed by fully virulent *Y. pestis* strains KIM and CO92 produced far more poly-GlcNAc in cells grown at 37 °C in broth cultures and on Congo red agar plates compared with the lower temperatures conducive to flea reproduction. We also show that there was no clear reduction in poly-GlcNAc in planktonic cells at 22^ ^°C and only a small reduction in biofilm on glass at this temperature. We do however observe that the *nagC* mutant is attenuated for biofilm on the surface of *C. elegans* at 22^ ^°C. This supports previous observations that the biotic worm cuticle is important in biofilm formation at this temperature [4], and may suggest that the presence of NagC is a contributing factor, especially given that NagC positively influences biofilm formation in *E. coli* AIEC LF82 [38] and curli fibre production that is also important for biofilm development [39]. Since *Yersinia* EPS contains poly-GlcNAc [7, 8] which is synthesised and transported by products of the *hms* locus [3, 10–16], and as we have previously reported that biofilm formation is QS dependent, our initial observations stimulated a more detailed examination of the role of NagC in *Yersinia *.

To investigate how the observations relating to poly-GlcNAc production might link to biofilm formation we conducted abiotic (glass) and biotic (*C. elegans*) surface biofilm assays. The loss of poly-GlcNAc and the reduction in biofilm on glass at 37^ ^°C in the *nagC* mutant may be a result of GlcNAc degradation and/or reduced *hms* expression and in addition, the considerable difference in *hms* expression in the *nagC* mutant at 37^ ^°C compared to 26^ ^°C may help to explain why the difference in poly-GlcNAc and biofilm at the lower temperature is also reduced. However, it is entirely feasible that temperature acts as an important environmental trigger for NagC functionality, initiating a differential response depending on the local conditions such as those found in an infection context. Although it is not possible to make a direct comparison at 37 °C because *C. elegans* infection assays cannot be carried out at this temperature, at 22^ ^°C the *nagC* mutant behaves differently in the *C. elegans* biofilm model when compared with planktonic cells and biofilms on glass, since biofilm formation is almost completely abolished on the worm cuticle.

We have previously reported that the double AHL synthase (*ypsI/ytbI*), AHL regulator (*ypsR/ytbR*) and motility mutants are attenuated for biofilm on the surface of *C. elegans* [23] and in comparison, when the *nagC* mutant was used to infect *C. elegans* the degree of attenuation was in excess of anything we had previously observed. Crucially adding back GlcNAc at a concentration of 5 mM was sufficient to restore biofilm to the levels observed in the parent, which suggested a link between biofilm formation and the activity of NagC. These data suggest that as with the observations at 37^ ^°C, the absence of NagC may trigger GlcNAc metabolism which reduces the amount available for poly-GlcNAc production and hence biofilm formation, possibly through the activities of NagC-dependent *hmsHFRS* expression. However, if an excess of GlcNAc is available there may be sufficient to overcome the metabolic consequences of an unregulated *nag* operon and so sufficient GlcNAc is again available for biofilm formation on *C. elegans*.

As QS in *Y. pseudotuberculosis* has previously been associated with aggregation, motility and Yop phenotypes [22] it was important to examine whether the *nagC* mutation affected these phenotypes. However, aggregation assays, semi-solid agar plate swimming motility assays and SDS-PAGE on supernatants induced for Yop secretion did not any reveal differences between the parent and the *nagC* mutant.

We then sought to investigate whether there is a regulatory relationship between NagC and *hms* and the promoter fusion data revealed that NagC positively activates the expression of *hmsHFRS* and so NagC represents an additional regulatory element involved in modulating the expression of *hms* to ensure that GlcNAc metabolism and biofilm formation are co-ordinately controlled as a function of prevailing environmental conditions. It is also possible that these systems influence peptidoglycan biosynthesis which is essential for survival and is where GlcNAc will be directed as a priority; similarly, LPS production may also be impacted.

The data presented here for NagC, coupled to our previous work linking QS to biofilms prompted us to examine the regulatory relationship between GlcNAc and the QS system. In *E. coli* NagC represses *nagE-nagBACD* and Miyashiro *et al*. [40] have shown that a similar GlcNAc-dependent NagC-mediated mechanism is essential for colonisation of the Hawaiian squid, *Euprymna scolopes* by *Vibrio fischeri* which, has a well defined AHL-dependent QS system regulating bioluminescence. To validate our promoter fusions and expression assays we therefore investigated whether control of the *Y. pseudotuberculosis* Nag operon was similar to that in *E. coli* where an increase in extracellular GlcNAc de-represses the *nag* operon [20, 24–27]. It is unsurprising therefore that that at 37^ ^°C in *Yersinia, nagC* expression increased with increasing GlcNAc concentration but was unresponsive when *nagC* was deleted. NagC is therefore a GlcNAc dependent autoregulator in *Y. pseudotuberculosis *, as it is in *E. coli *.

Kimyon *et al*. [41] reported that GlcNAc inhibits transcription of genes upregulated by AHL binding to *V. fischeri* LuxR*, Pseudomonas aeruginosa* LasR*,* and *Chromobacterium violaceum* CviR as well as affecting AHL-dependent phenotypes. In comparison, using *lux*-based reporter fusion technology we have extended these observations to show a regulatory link between NagC and QS which reveals that GlcNAc metabolism in the form of NagC-mediated regulation is linked to *ytbI* and *ypsI*. This suggests that in *Yersinia* NagC not only regulates GlcNAc metabolism in response to fluctuations in GlcNAc concentration but has a wider regulatory role linked to population density. As a member of the ROK protein family NagC is regarded as a regulator and appears to carry out this role in negatively regulating *ytbI* and *ypsI* expression. It is also evident that *ytbI* and *ytbR* expression are governed to some extent by the extracellular GlcNAc concentration. The GlcNAc-dependent reduction in expression that we observe for *ytbI* in the parent but not the *nagC* mutant suggests that there is a requirement for both NagC and GlcNAc that, in combination, will repress expression by acting at the *ytbI* promoter. Expression of *ytbR* appears to be GlcNAc-dependent but NagC-independent since both the parent and the *nagC* mutant show a comparable reduction in GlcNAc-dependent activity. To our knowledge there are no reports of GlcNAc acting directly on a DNA target but it is possible that as a regulator, YtbR has the potential to bind GlcNAc-6-P independently of NagC to regulate its own expression and possibly other target genes. It is difficult to make similar inferences about the YpsRI response to GlcNAc since there was little or no effect on expression.

Given that AHL synthase gene expression was elevated in the *nagC* mutant (Fig. 6a) we may have expected increased AHL production in the mutant when compared with the parent. However, contrary to these expectations, LC-ESI MS/MS [32] revealed that rather than increasing AHL levels, the *nagC* mutation resulted in reduced AHL levels. These were fully restored in the genetically complemented *nagC* mutant. Since the *ypsI* and *ytbI lux* promoter fusions have previously been extensively validated [20], and as *nagC* expression behaved as expected (consistent with that previously reported for *E. coli* [24–26] where a linear increase in expression in response to increasing GlcNAc concentrations was observed and with little or no response in the *nag*C mutant), an alternative explanation is required. This could involve mRNA transcript processing or inhibition of translation of the AHL synthase genes in the *nagC* mutant. Alternatively, a homeostatic control mechanism(s) preventing AHL concentrations increasing beyond the physiological concentrations required would ensure that key cellular metabolites such as *S*-adenosylmethionine (SAM) and acyl-CoA intermediates that are substrates for the YpsI and YtbI AHL synthases are not exhausted. Such a mechanism has been described in *P. aeruginosa* in which the transcriptional regulator RsaL represses expression of both the synthase (*lasI*) and the response regulator (*lasR*) genes so limiting *N*-(3-oxododecanoyl) homoserine lactone (3-oxo-C12-HSL) production [42]. In an *rsaL* mutant, 3-oxo-C12-HSL concentrations continue to increase substantially with growth rather than reaching a plateau. Alternatively, AHL homoeostasis could be achieved via the regulated expression or activity of AHL-degrading enzymes such as lactonase, acylases or oxido-reductases whose expression may be under the control of NagC [43], although to our knowledge none have yet been identified in *Yersinia* spp. It is also noteworthy that in contrast to most LuxR/I-based QS systems, for *Y. pseudotuberculosis* and *Yersinia enterocolitica,* the addition of exogenous AHLs to AHL synthase mutants does not restore QS phenotypes despite the AHLs being confirmed (through reporter assays and by using tritiated AHLs) as entering the cells [22, 31]. This is reminiscent of the *Chromobacterium violaceum* CV026 *cviI* mutant in which violacein pigment production cannot be restored with exogenous AHLs. Restoration of violacein required a second mutation in a repressor in order to enable the *cviI* mutant to respond [44].

However, whatever the mechanism that leads to reduced AHL levels despite the increased transcription of *ypsI* and *ytbI*, it clearly involves NagC either directly or indirectly. Further experimental work will be required to unravel the molecular basis of these observations.

We have also uncovered a reciprocal regulatory relationship between NagC and the QS system since at 26^ ^°C and 37^ ^°C *nagC* expression is reduced in the *ypsI, ypsR* and *ytbR* systems indicating that population-wide monitoring can activate *nagC* expression. However, *nagC* expression remains unaffected in the *ytbI* mutant. As a signal synthase YtbI is responsible for the synthesis of a range of AHLs as well as several which are also produced via YpsI [32]. Given that the full repertoire of YpsI-derived AHLs are available in the *ypsI* mutant due to the synthase activity of YtbI, it is interesting that mutation of *ypsI* rather than *ytbI* has an effect on *nagC* expression. To our knowledge there are no reports of an AHL synthase fulfilling an additional role as an activator/repressor autonomously and therefore this observation remains intriguing.

**Fig. 9.**
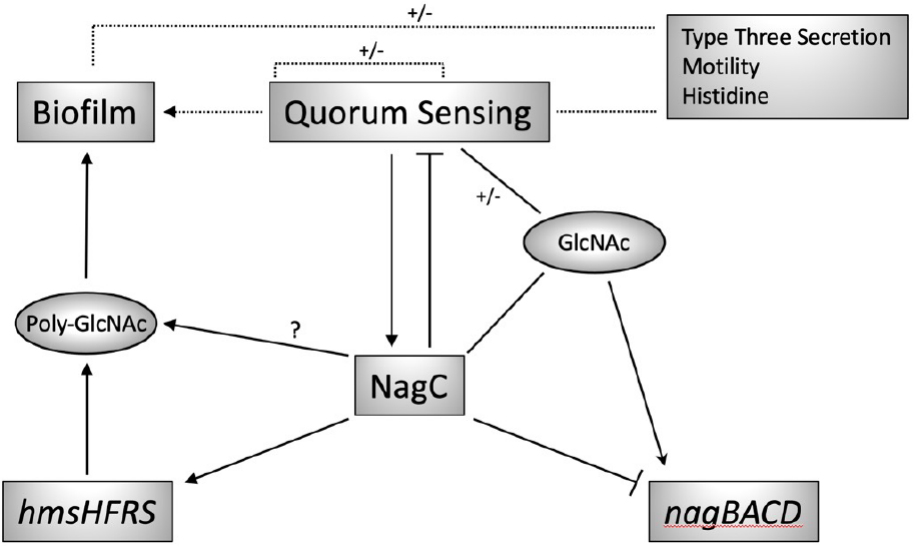
Model to show the relationship between NagC, QS and GlcNAc metabolism in *Y. pseudotuberculosis *. NagC positively and negatively regulates the expression of the *nag* operon in a GlcNAc dependent manner. There is a reciprocal activator/repressor regulatory relationship between QS and NagC and NagC positively regulates biofilm formation, either directly or through the positive activation of *hmsHFRS* expression. The dashed lines represent our previous work defining the interdependent regulatory relationships between QS, biofilm, type three secretion, motility and histidine metabolism [20, 21, 32].

It is evident from our findings that the availability of GlcNAc and NagC-mediated GlcNAc catabolism can impact on the production of poly-GlcNAc and biofilm on biotic and abiotic surfaces. This is linked to the regulatory network involving the *nag*, *hms* and QS operons where there are reciprocal regulatory relationships between the systems which presumably enables a population-wide response to fluctuations in available GlcNAc. Conceptually it seems logical that the metabolism of such an abundant carbon source such as GlcNAc, which is integral to the biofilm lifestyle would be linked to QS to ensure that a bacterial population can coordinate its efforts to metabolize the available substrate to accommodate fluctuating environmental challenges. Fig. 9 presents a model to describe the findings presented in this manuscript and highlights the key role NagC plays in regulating its own expression, the *nagBACD* operon, QS, *hmsHFRS* and biofilm formation. Fig. 9 also shows that NagC forms part of a wider regulatory network including type three secretion, motility and histidine metabolism in *Y. pseudotuberculosis* [22, 23, 33].

We have uncovered a sophisticated regulatory system based around QS, T3S, biofilms and motility in *Y. pseudotuberculosis* which couples population sensing with environmental cues to facilitate adaptation in a changing environment. This study indicates that NagC and *hms* are additional components within this elegant system providing the bacterial population with the capacity to monitor and metabolise GlcNAc as part of this wider interconnected regulatory network.

## Supplementary Data

Supplementary material 1

## References

[1] Stoodley P, Sauer K, Davies DG, Costerton JW (2002). Biofilms as complex differentiated communities. Annu Rev Microbiol.

[2] McNally A, Thomson NR, Reuter S, Wren BW (2016). “Add, stir and reduce”: *Yersinia* spp. as model bacteria for pathogen evolution. Nat Rev Microbiol.

[3] Darby C, Hsu JW, Ghori N, Falkow S (2002). *Caenorhabditis elegans*: plague bacteria biofilm blocks food intake. Nature.

[4] Joshua GWP, Karlyshev AV, Smith MP, Isherwood KE, Titball RW (2003). A *Caenorhabditis elegans* model of *Yersinia* infection: biofilm formation on a biotic surface. Microbiology.

[5] Bacot AW, Martin CJ (1914). LXVII. Observations on the mechanism of the transmission of plague by fleas. J Hyg.

[6] Bacot AW (1915). LXXXI. Further notes on the mechanism of the transmission of plague by fleas. J Hyg.

[7] Tan L, Darby C (2004). A movable surface: formation of *Yersinia* sp. biofilms on motile *Caenorhabditis elegans*. J Bacteriol.

[8] Bobrov AG, Kirillina O, Forman S, Mack D, Perry RD (2008). Insights into *Yersinia pestis* biofilm development: topology and co-interaction of Hms inner membrane proteins involved in exopolysaccharide production. Environ Microbiol.

[9] Hinnebusch BJ, Erickson DL (2008). *Yersinia pestis* biofilm in the flea vector and its role in the transmission of plague. Bacterial biofilms.

[10] Perry RD, Pendrak ML, Schuetze P (1990). Identification and cloning of a hemin storage locus involved in the pigmentation phenotype of *Yersinia pestis*. J Bacteriol.

[11] Hinnebusch BJ, Perry RD, Schwan TG (1996). Role of the *Yersinia pestis* hemin storage (hms) locus in the transmission of plague by fleas. Science.

[12] Jarrett CO, Deak E, Isherwood KE, Oyston PC, Fischer ER (2004). Transmission of *Yersinia pestis* from an infectious biofilm in the flea vector. J Infect Dis.

[13] Ren G-X, Yan H-Q, Zhu H, Guo X-P, Sun Y-C (2014). HmsC, a periplasmic protein, controls biofilm formation via repression of HmsD, a diguanylate cyclase in *Yersinia pestis*. Environ Microbiol.

[14] Fang N, Qu S, Yang H, Fang H, Liu L (2014). HmsB enhances biofilm formation in *Yersinia pestis*. Front Microbiol.

[15] Jones HA, Lillard JW, Perry RD (1999). HmsT, a protein essential for expression of the haemin storage (Hms+) phenotype of *Yersinia pestis*. Microbiology.

[16] Kirillina O, Fetherston JD, Bobrov AG, Abney J, Perry RD (2004). HmsP, a putative phosphodiesterase, and HmsT, a putative diguanylate cyclase, control Hms-dependent biofilm formation in *Yersinia pestis*. Mol Microbiol.

[17] Bobrov AG, Kirillina O, Ryjenkov DA, Waters CM, Price PA (2011). Systematic analysis of cyclic di-GMP signalling enzymes and their role in biofilm formation and virulence in *Yersinia pestis*. Mol Microbiol.

[18] Sun Y-C, Koumoutsi A, Jarrett C, Lawrence K, Gherardini FC (2011). Differential control of *Yersinia pestis* biofilm formation in vitro and in the flea vector by two c-di-GMP diguanylate cyclases. PLoS One.

[19] Bobrov AG, Kirillina O, Perry RD (2005). The phosphodiesterase activity of the HmsP EAL domain is required for negative regulation of biofilm formation in *Yersinia pestis*. FEMS Microbiol Lett.

[20] Plumbridge JA (1991). Repression and induction of the nag regulon of *Escherichia coli* K-12: the roles of nagC and nagA in maintenance of the uninduced state. Mol Microbiol.

[21] Winzer K, Hardie KR, Williams P (2002). Bacterial cell-to-cell communication: sorry, can’t talk now - gone to lunch!. Curr Opin Microbiol.

[22] Atkinson S, Chang C-Y, Patrick HL, Buckley CMF, Wang Y (2008). Functional interplay between the *Yersinia pseudotuberculosis* YpsRI and YtbRI quorum sensing systems modulates swimming motility by controlling expression of flhDC and fliA. Mol Microbiol.

[23] Atkinson S, Goldstone RJ, Joshua GWP, Chang C-Y, Patrick HL (2011). Biofilm development on *Caenorhabditis elegans* by *Yersinia* is facilitated by quorum sensing-dependent repression of type III secretion. PLoS Pathog.

[24] Plumbridge J, Kolb A (1993). DNA loop formation between Nag repressor molecules bound to its two operator sites is necessary for repression of the nag regulon of *Escherichia coli* in vivo. Mol Microbiol.

[25] Plumbridge J (1995). Co-ordinated regulation of amino sugar biosynthesis and degradation: the NagC repressor acts as both an activator and a repressor for the transcription of the glmUS operon and requires two separated NagC binding sites. EMBO J.

[26] Titgemeyer F, Reizer J, Reizer A, Saier MH (1994). Evolutionary relationships between sugar kinases and transcriptional repressors in bacteria. Microbiology.

[27] Plumbridge JA (1990). Induction of the nag regulon of *Escherichia coli* by N-acetylglucosamine and glucosamine: role of the cyclic AMP-catabolite activator protein complex in expression of the regulon. J Bacteriol.

[28] Datsenko KA, Wanner BL (2000). One-step inactivation of chromosomal genes in *Escherichia coli* K-12 using PCR products. Proc Natl Acad Sci U S A.

[29] Derbise A, Lesic B, Dacheux D, Ghigo JM, Carniel E (2003). A rapid and simple method for inactivating chromosomal genes in *Yersinia*. FEMS Immunol Med Microbiol.

[30] Choi K-H, Gaynor JB, White KG, Lopez C, Bosio CM (2005). A Tn7-based broad-range bacterial cloning and expression system. Nat Methods.

[31] Ng Y-K, Grasso M, Wright V, Garcia V, Williams P (2018). The quorum sensing system of *Yersinia enterocolitica* 8081 regulates swimming motility, host cell attachment, and virulence plasmid maintenance. Genes.

[32] Ortori CA, Atkinson S, Chhabra SR, Cámara M, Williams P (2007). Comprehensive profiling of N-acylhomoserine lactones produced by *Yersinia pseudotuberculosis* using liquid chromatography coupled to hybrid quadrupole-linear ion trap mass spectrometry. Anal Bioanal Chem.

[33] Joshua GWP, Atkinson S, Goldstone RJ, Patrick HL, Stabler RA (2014). Genome-wide evaluation of the interplay between *Caenorhabditis elegans* and *Yersinia pseudotuberculosis* during in vivo biofilm formation. Infect Immun.

[34] Qazi SNA, Rees CED, Mellits KH, Hill PJ (2001). Development of gfp vectors for expression in *Listeria monocytogenes* and other low G+C gram positive bacteria. Microb Ecol.

[35] Lavander M, Ericsson SK, Bröms JE, Forsberg A (2006). The twin arginine translocation system is essential for virulence of *Yersinia pseudotuberculosis*. Infect Immun.

[36] Yoong P, Cywes-Bentley C, Pier GB (2012). Poly-N-acetylglucosamine expression by wild-type *Yersinia pestis* is maximal at mammalian, not flea, temperatures. mBio.

[37] Plumbridge J (2001). DNA binding sites for the Mlc and NagC proteins: regulation of nagE, encoding the N-acetylglucosamine-specific transporter in *Escherichia coli*. Nucleic Acids Res.

[38] Sicard J-F, Vogeleer P, Le Bihan G, Rodriguez Olivera Y, Beaudry F (2018). N-Acetyl-glucosamine influences the biofilm formation of *Escherichia coli*. Gut Pathog.

[39] Barnhart MM, Lynem J, Chapman MR (2006). GlcNAc-6P levels modulate the expression of Curli fibers by *Escherichia coli*. J Bacteriol.

[40] Miyashiro T, Klein W, Oehlert D, Cao X, Schwartzman J (2011). The N-acetyl-D-glucosamine repressor NagC of *Vibrio fischeri* facilitates colonization of Euprymna scolopes. Mol Microbiol.

[41] Kimyon Ö, Ulutürk ZI, Nizalapur S, Lee M, Kutty SK (2016). N-Acetylglucosamine inhibits LuxR, LasR and CviR based quorum sensing regulated gene expression levels. Front Microbiol.

[42] Rampioni G, Schuster M, Greenberg EP, Bertani I, Grasso M (2007). RsaL provides quorum sensing homeostasis and functions as a global regulator of gene expression in *Pseudomonas aeruginosa*. Mol Microbiol.

[43] Torres M, Uroz S, Salto R, Fauchery L, Quesada E (2017). HqiA, a novel quorum-quenching enzyme which expands the AHL lactonase family. Sci Rep.

[44] McClean KH, Winson MK, Fish L, Taylor A, Chhabra SR (1997). Quorum sensing and *Chromobacterium violaceum*: exploitation of violacein production and inhibition for the detection of N-acylhomoserine lactones. Microbiology.

