# Supplementary material for: Reciprocal regulation of NagC and quorum sensing systems and their roles in hmsHFRS expression and biofilm formation in Yersinia pseudotuberculosis
Source: Microbiology (Reading). 2023 Oct 10;169(10):001397. doi: 10.1099/mic.0.001397 (PMC10634360; doi:10.1099/mic.0.001397)
Supplement: Supplementary material 1 [file mic-169-1397-s001.pdf]

Supplementary Material

Reciprocal regulation of NagC and Quorum Sensing systems and their roles in *hmsHFRS* expression and biofilm formation in *Yersinia pseudotuberculosis*.

Anja Wiechmann, Vanina Garcia, Linzy Elton[†], Paul Williams and Steve Atkinson*

Biodiscovery Institute, School of Life Sciences, University of Nottingham, Nottingham, NG7 2RD, United Kingdom.

[†]Division of Infection and Immunity, University College, London, WC1E 6BT. United Kingdom.

* Corresponding author

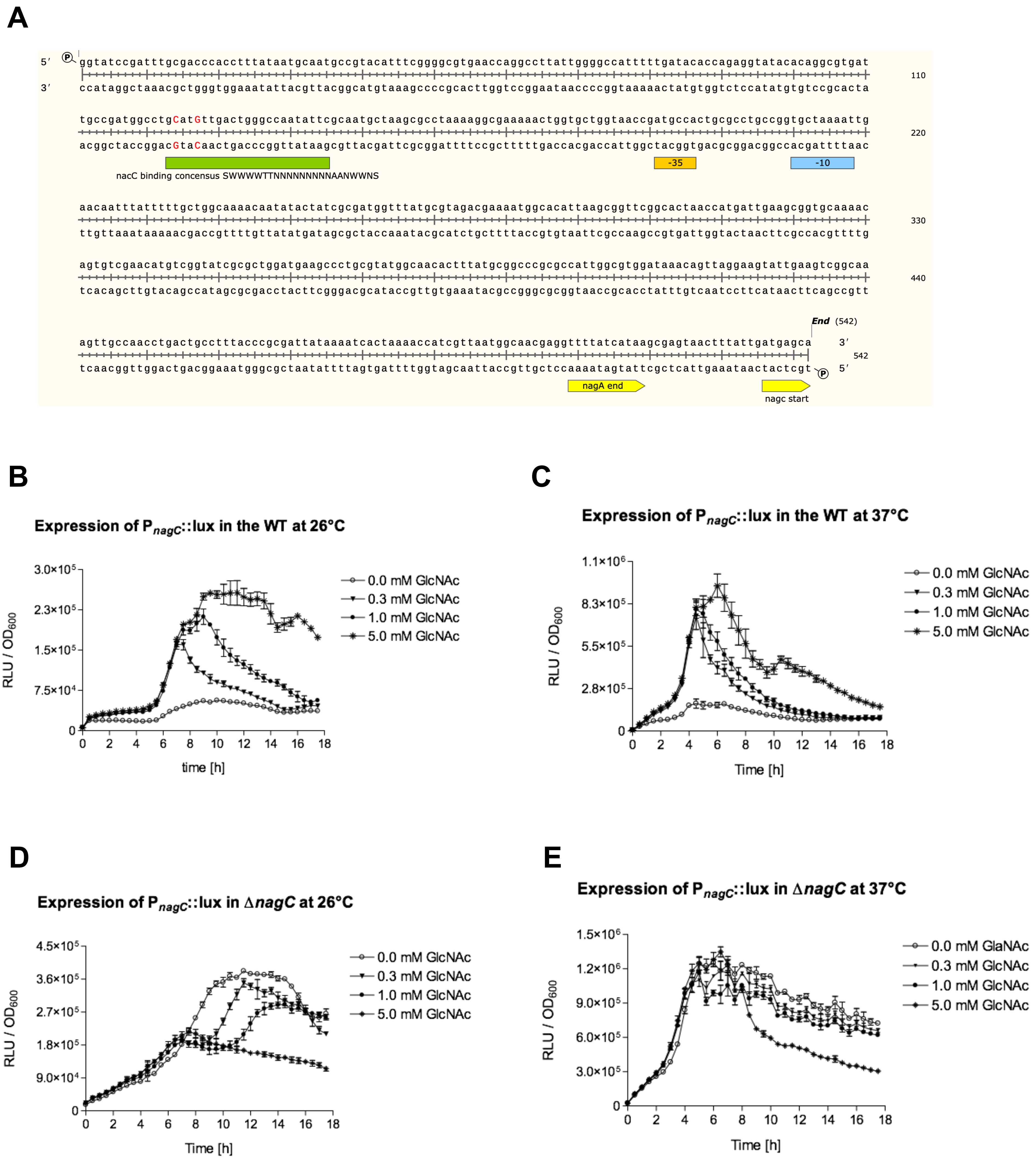

Fig S1. *Y. pseudotuberculosis nagC* possesses a putative *nagC* binding site (green box) in the *nagC* promoter region, with only 2 mismatches (red) to the consensus (**A**). Full expression profiles of NagC in the *Y. pseudotuberculosis* parent (WT) and *nagC* mutant with increasing concentrations of GlCNAc at 26°C and 37°C (**B**).

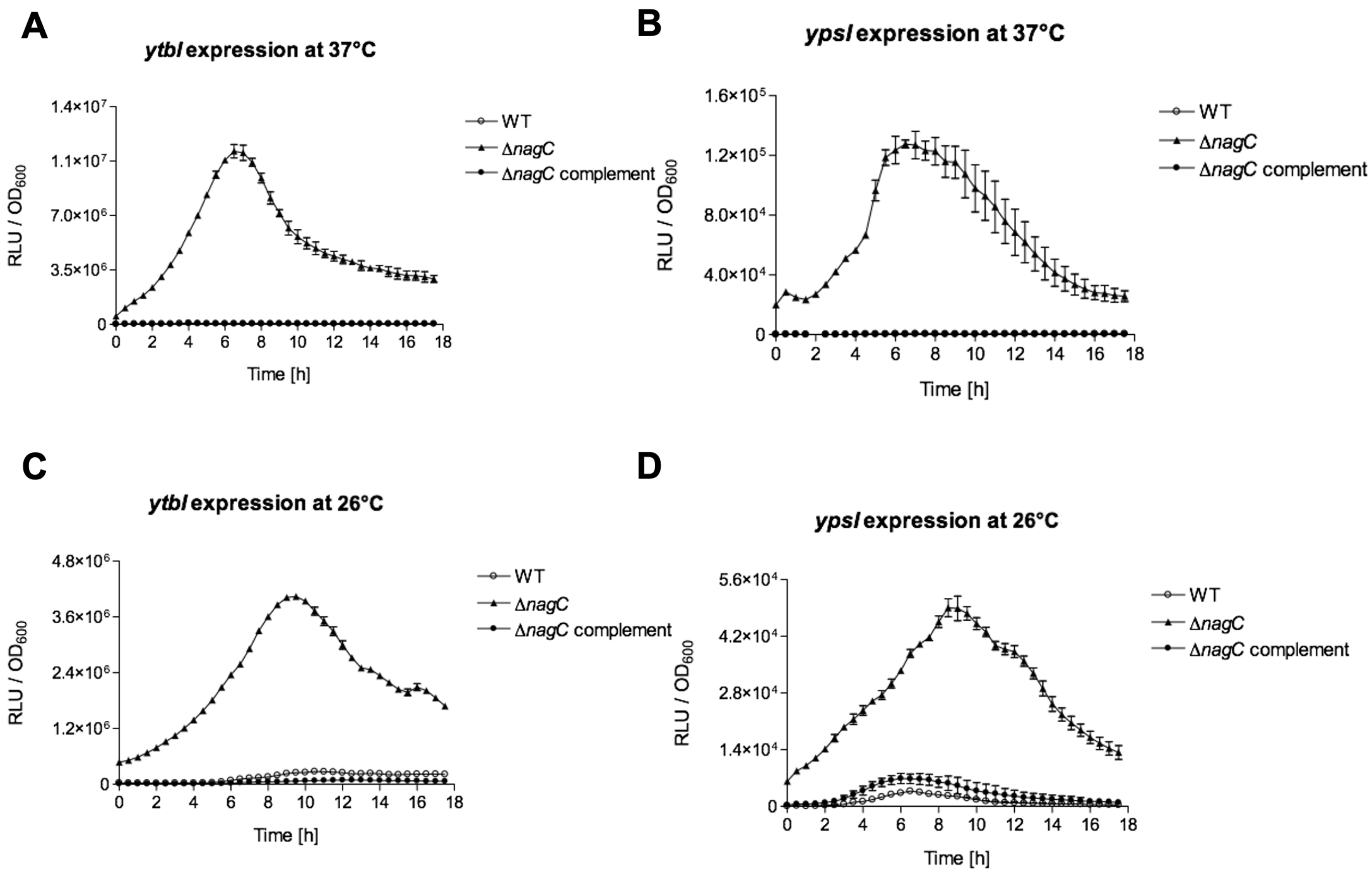

Fig S2. Full expression profiles of *ytbI* and *ypsI* in the *Y. pseudotuberculosis* parent (WT), *nagC* mutant and complement at 26°C and 37°C (**A-D**).

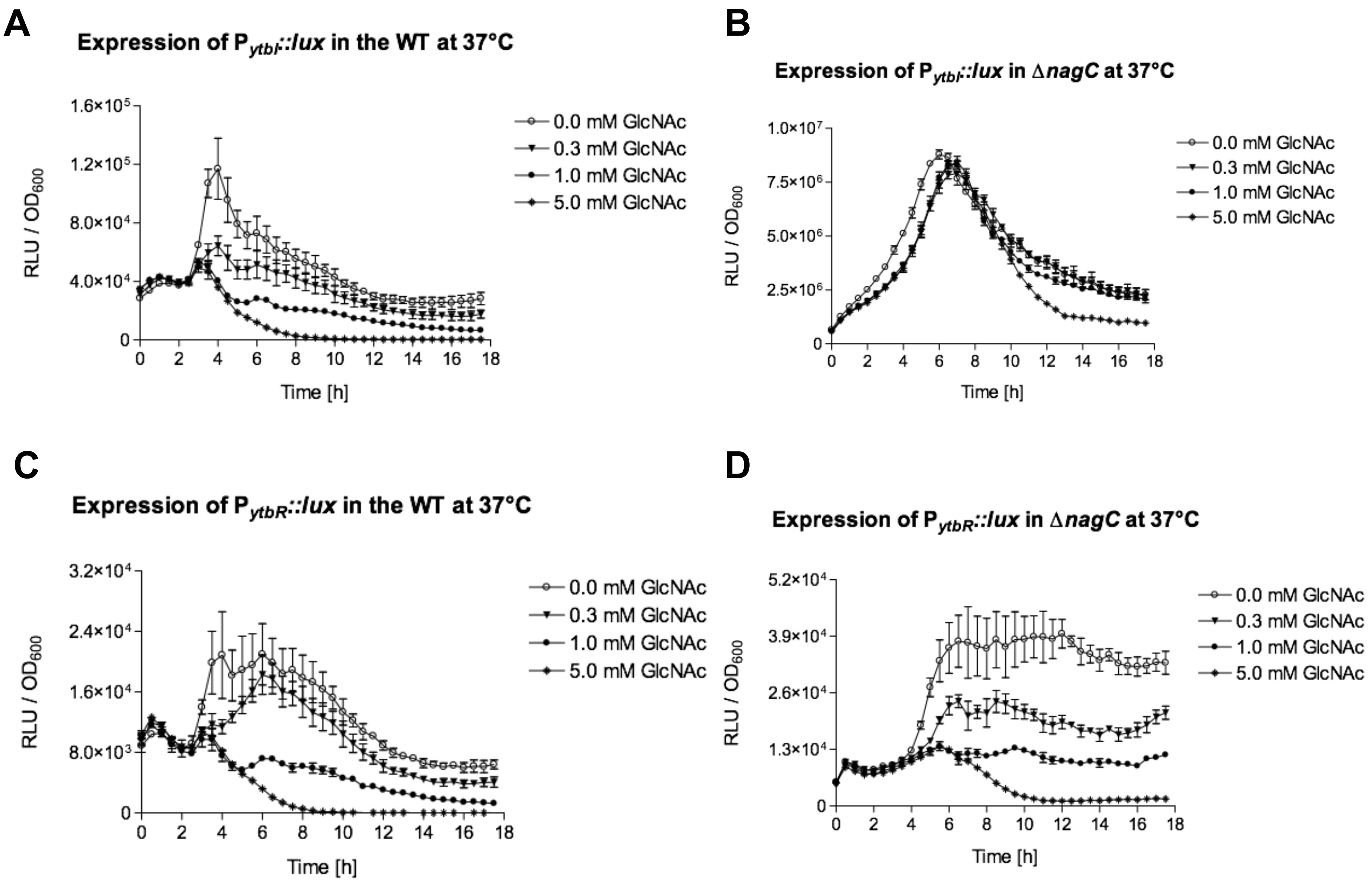

Fig S3. Full expression profiles of *ytbI* and *ytbR* in the *Y. pseudotuberculosis* parent (WT) and *nagC* mutant in increasing GlcNAc concentrations at 37°C (**A-D**).

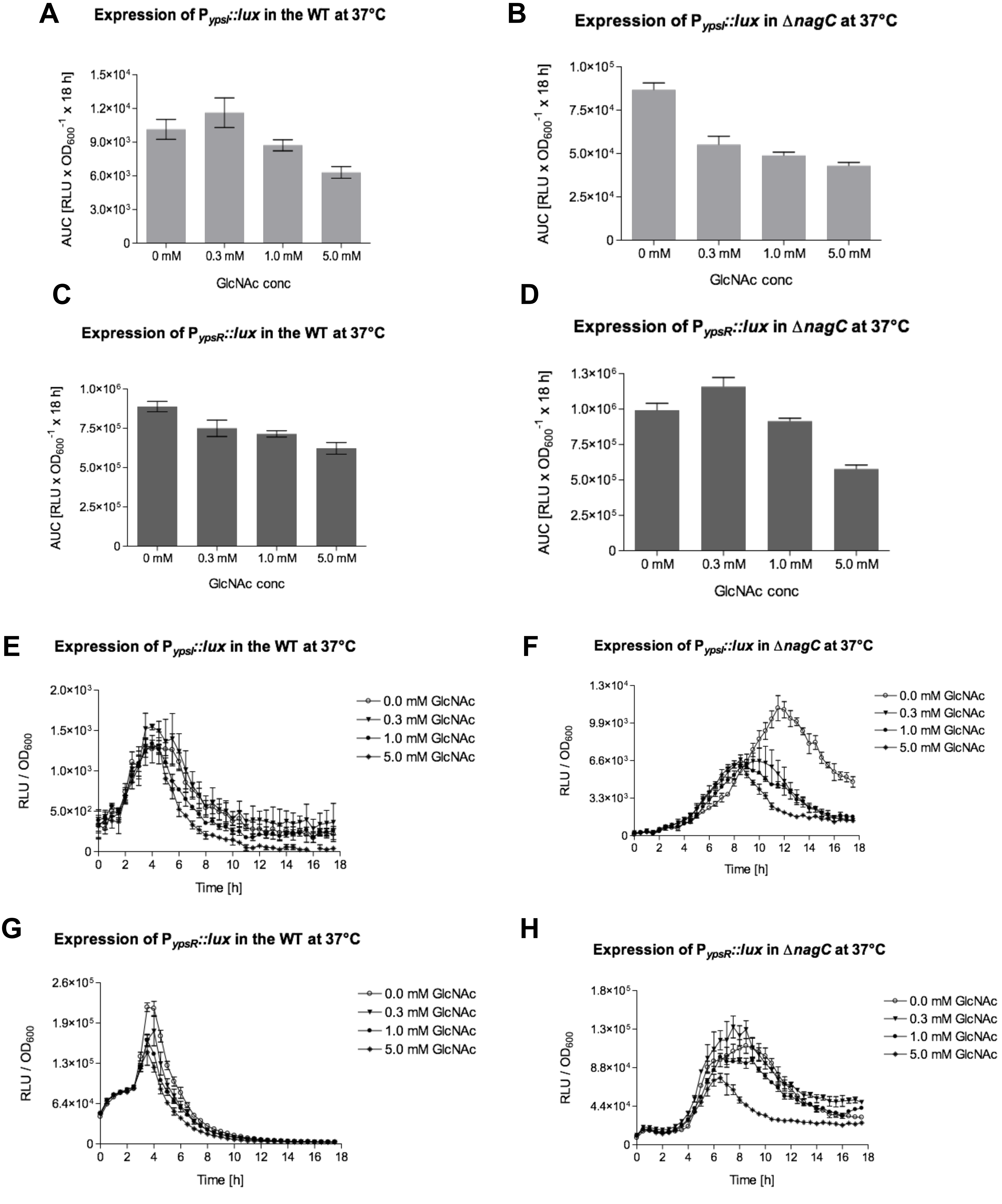

Fig S4. Expression as AUC of *ypsI* and *ypsR* in the parent (WT) and *nagC* mutant with increasing GlcNAc concentrations at 37°C (**A-D**). The full expression profiles reveal the profiles of the curves over 18 h (**E-H**).

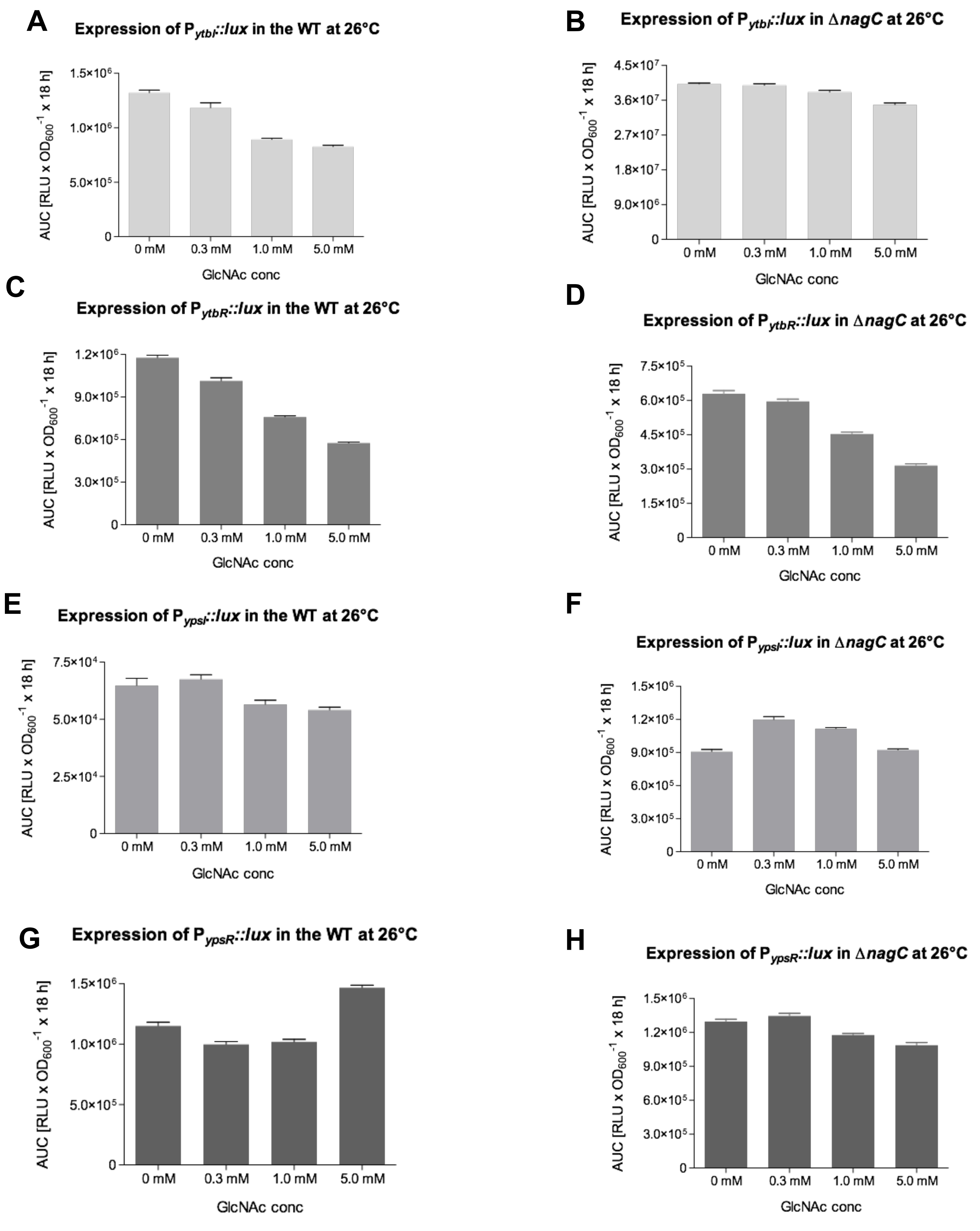

Fig S5. Expression as AUC of *ytbI*, *ytbR*, *ypsI* and *ypsR* in the parent (WT) and *nagC* mutant with increasing GlcNAc concentrations at 26°C (**A-H**).

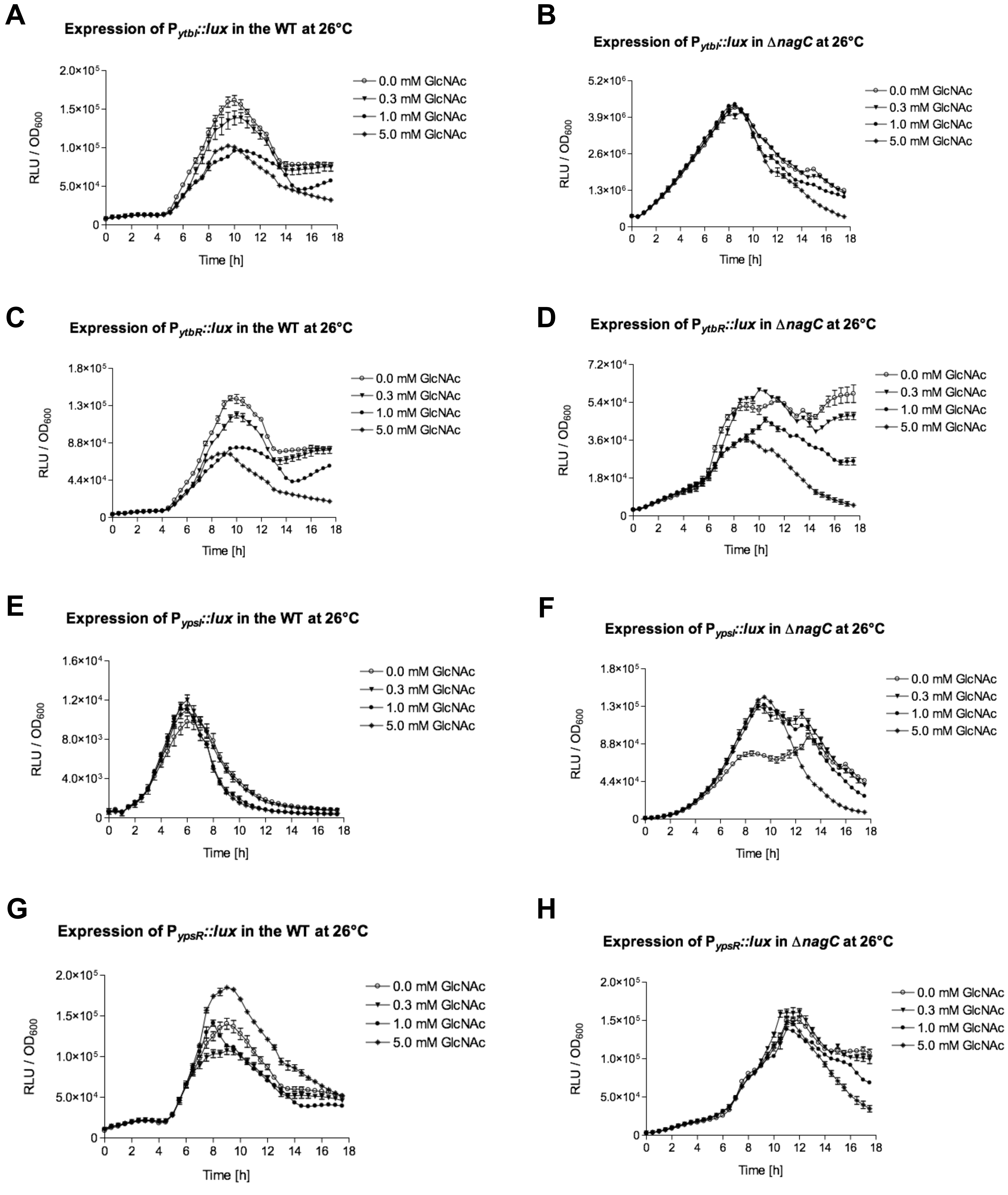

Fig S6. Full expression profile of *ytbI*, *ytbR*, *ypsI* and *ypsR* in the parent (WT) and *nagC* mutant with increasing GlcNAc concentrations at 26°C (**A-H**). Use in conjunction with **Fig. S5**.

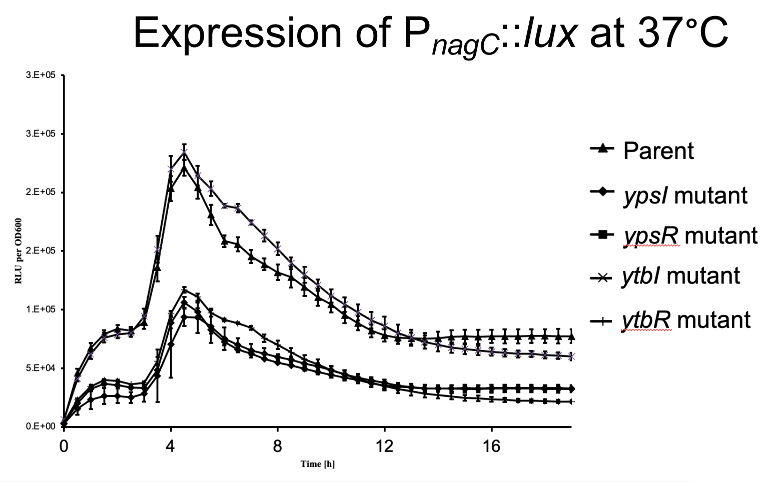

Fig S7. Full expression profile of *nagC* in the parent and *ytbI*, *ytbR*, *ypsI* and *ypsR* quorum sensing mutants.

Table S1. Plasmids

Name	Description	Source
pUC4K	Source of kanamycin resistance cassette	Pharmacia
pAJD434	λ-red recombinase plasmid inducible with arabinose (Tm^r)	[1]
pGEM-T Easy	$Ampicillin^r$ PCR product cloning vector	Promega
pUC18R6KT::Tc mini Tn*7*	Source of mini Tn*7* transposon	[2]
pTNS2	pUX-BF13 *Ori*; mini-Tn*7* helper plasmid, encodes TnsABC +D transposase complex to catalyze high frequency insertion (Amp^r)	[2]
pDM4	Suicide vector containing *mob*RK2, *ori*R6K and *sacBR* (Cm^r)	[3]
pBlue*lux*	pBluescript II KS+ Vector containing the *luxCDABE* operon (Amp^r)	[4]
pHG327	Low copy number cloning vector (Amp^r)	[5]
pSB2020	Gfp vector for labelling (Amp^r)	[6]
pHP276	pKNG101 suicide vector containing a promoter::*lux* fusion of *ypsI*	[4]
pHP277	pKNG101 suicide vector containing a promoter::*lux* fusion of *ypsR*	[4]
pHP278	pKNG101 suicide vector containing a promoter::*lux* fusion of *ytbI*	[4]
pHP279	pKNG101 suicide vector containing a promoter::*lux* fusion of *ytbR*	[4]
pHG::$P_{hms::lux}$	pHG327 containing the *hmsHFRS* promoter fusion	This study
pDM4::$P_{nagC::lux}$	pDM4 Suicide vector containing a promoter::*lux* fusion of *nagC* (Cm^r)	This study
pAW1	Tn7 suicide vector containing a parental copy of *nagC* for complementation.	This study

1. **Derbise A, Lesic B, Dacheux D, Ghigo J-M, Carniel E**. A rapid and simple method for inactivating chromosomal genes in Yersinia. *Fems Immunology and Medical Microbiology* 2003;38(2):113 - 116.

2. **Choi KH, Gaynor JB, White KG, Lopez C, Bosio CM et al.** A Tn7-based broad-range bacterial cloning and expression system. *Nat Methods* 2005;2(6):443-448.

3. **O'Toole R, Milton DL, Wolf-Watz H**. Chemotactic motility is required for invasion of the host by the fish pathogen Vibrio anguillarum. *MolMicrobiol* 1996;19(3):625 - 637.

4. **Atkinson S, Chang CY, Patrick HL, Buckley CMF, Wang Y et al.** Functional interplay between the Yersinia pseudotuberculosis YpsRI and YtbRI quorum sensing systems modulates swimming motility by controlling expression of flhDC and fliA. *MolMicrobiol* 2008;69(1):137-151.

5. **Stewart GSAB, Lubinskymink S, Jackson CG, Cassel A, Kuhn J**. pHG165 - a pBR322 copy number derivative of pUC8 for cloning and expression. *Plasmid PMID - 3012611* 1986;15(3):172 - 181.

6. **Qazi SN, Rees CE, Mellits KH, Hill PJ**. Development of gfp Vectors for Expression in Listeria monocytogenes and Other Low G+C Gram Positive Bacteria. *Microb Ecol* 2001;41(4):301-309.

Table S2. Primers

Name	Sequence (5’-3’)	Restriction Site	Source
nagC-ko-F	GGCGGACAAGCACAAATTGGTAACGTGGATCTGGTAAAACAACTCAAGGAGAAAGCCACGTTGTGTCTCAA		This study
nagC-ko-R	AGTTTCTAGCAAACGTTGCAGCAAGACACCATTGAGCATAGCGCGCTTAGCTTAGAAAAACTCATCGAGCAT		This study
nagC-comp-F	**CTCGAG**CGGTATCCGATTTGCGACC	XhoI	This study
nagC-comp-R	**GGATCC**GGTCACTATAACATGGCTGTGG	BamHI	This study
nagC-*lux*-F	**CTCGAG**CGGTATCCGATTTGCGACC	XhoI	This study
nagC-*lux*-R	**GGATCCGGTACC**CCTCGTTGCCATTAACGATGG	KpnI, BamHI	This study
VG-Phms-F2-ApaI	ATTTCT**GGGCCC**TTCATTGTATCGTAGCCCGGATAAAAC	ApaI	This study
VG-Phms-R-XhoI	GCGTTAT**CTCGAG**TATAACCCTTAAGCCAGCAACTTGTTTGA	XhoI	This study
